# Mesoscale Anisotropy in Porous Media Made of Clay Minerals. A Numerical Study Constrained by Experimental Data

**DOI:** 10.3390/ma11101972

**Published:** 2018-10-13

**Authors:** Thomas Dabat, Arnaud Mazurier, Fabien Hubert, Emmanuel Tertre, Brian Grégoire, Baptiste Dazas, Eric Ferrage

**Affiliations:** CNRS (Centre National de la Recherche Scientifique), Université de Poitiers, UMR 7285 IC2MP-Hydrasa, 5 rue Albert Turpain, Bâtiment B8, TSA 51106, 86073 Poitiers CEDEX 9, France; arnaud.mazurier@univ-poitiers.fr (A.M.); fabien.hubert@univ-poitiers.fr (F.H.); emmanuel.tertre@univ-poitiers.fr (E.T.); brian.gregoire@univ-poitiers.fr (B.G.); baptiste.dazas@univ-poitiers.fr (B.D.)

**Keywords:** clay minerals particles, orientational anisotropy, granular systems, disk packing, X-Ray microtomography, mesoscale simulation

## Abstract

The anisotropic properties of clay-rich porous media have significant impact on the directional dependence of fluids migration in environmental and engineering sciences. This anisotropy, linked to the preferential orientation of flat anisometric clay minerals particles, is studied here on the basis of the simulation of three-dimensional packings of non-interacting disks, using a sequential deposition algorithm under a gravitational field. Simulations show that the obtained porosities fall onto a single master curve when plotted against the anisotropy value. This finding is consistent with results from sedimentation experiments using polytetrafluoroethylene (PTFE) disks and subsequent extraction of particle anisotropy through X-ray microtomography. Further geometrical analyses of computed porous media highlight that both particle orientation and particle aggregation are responsible of the evolution of porosity as a function of anisotropy. Moreover, morphological analysis of the porous media using chord length measurements shows that the anisotropy of the pore and solid networks can be correlated with particle orientation. These results indicate that computed porous media, mimicking the organization of clay minerals, can be used to shed light on the anisotropic properties of fluid transfer in clay-based materials.

## 1. Introduction

The understanding of textural properties of natural porous media composed of clay minerals is of prime importance, owing to the key role of these minerals in retention and mobility of various resources (water, gas) and pollutants in natural environments such as soils and rocks [1,2,3,4] as well as for industrial application such as clay liners in civil engineering [5,6,7,8,9,10]. Crystal structure of clay minerals comprises one or two tetrahedral sheets (with mainly Si, Al) and one octahedral sheet (with Al, Mg, Fe, Li, etc.). At the molecular scale, exchange capacities governing reactivity of clay minerals result from negative charges created by isomorphic substitutions either in the tetrahedral and/or octahedral sheets balanced by cations [11].

Owing to their low aspect ratio (particle thickness to length ratio) coupled with large surface areas [12,13,14,15], clay minerals organization can be conveniently modelled with flat ellipsoids, disks, or elliptic disk-shaped particles [16,17,18,19]. Textural properties and associated preferential orientation of clay particles have substantial influence on the pore network morphology, such as pore size, tortuosity, and constrictivity impacting the directional dependence of fluid transfer [19,20,21,22,23]. In addition, the orientation of particles is inherited from initial geological conditions and may be further affected by compaction and shear forces [24,25,26].

Imaging techniques such as Transmission Electron Microscopy (TEM), Scanning Electron Microscopy (SEM) or tomography represent efficient methods to characterize the organization of clay minerals particles [4,20,27,28,29,30] especially for the in situ analysis of size and shape of pores. Given the multiscale nature of the clay-based materials, the spatial resolution needed to cover a wide range of porosities is still difficult to reach, especially for the smallest pore sizes. Three-dimensional (3D) particle packing simulation represents a powerful methodology for overcoming experimental limitations. As far as swelling clay minerals (i.e., smectite) are concerned, previous numerical works studied individual layers aggregation process by taking into account molecular interaction forces to derive particle formation mechanism [17,31,32,33]. For other types of platy-shaped clay minerals, thick particles are generally composed of a large number of firmly stacked individual layers. In such a case, the contribution from cohesive forces between particles is reduced and existing numerical works have mainly focused on the final organization of the porous medium and on the analysis of the mutual arrangements of grains in the packing [18,34,35]. In this latter case, and based on the sequential deposition algorithm from Coelho et al. [34], Ferrage et al. [18] recently showed that particle simulations considering non-interacting flat elliptic disks were able to reproduce different parameters measured on porous media composed of vermiculite particles, such as distribution of size, shape, and orientation of particle [36]. Moreover, this work also showed that both porosity and the degree of anisotropy in particle orientation have to be considered for anisometric particles.

In the present study, simulations of 3D disk packings with different anisotropy degrees are computed to better understand the geometrical properties of granular systems made of anisometric particles. While not attempting to reproduce the complexity of natural sedimentation processes, these simulations remain a good model for the analysis of preferential orientation of packed clay minerals particles. In the first section, the evolution of simulated porosity as a function of anisotropy is analyzed and validated against experimental data, provided by X-ray microtomography. Validation of simulations by experiments then allows, in the second section, the use of the computational results for a more detailed analysis of geometrical properties of these anisotropic granular systems.

## 2. Materials and Methods

### 2.1. Simulation of 3D Disk Packings

The simulated 3D disk packings were obtained using a sequential deposition algorithm of particles in a square simulation box of width *w* under a gravitational field [18,34]. As previously mentioned [18,34], such type of simulation does not reproduce the sedimentation process, but rather allows for generating grain packings with a wide range of anisotropic properties in particles orientations. According to this algorithm, the bottom of the simulation box (at *z* = 0) is considered rigid. Disks are introduced at the top of the box with a given initial angle. The particules then fall down until one or several contact points are detected with either the bottom of the box or the bed of settled particles. Once a contact is detected, the disk settles using the steepest descent method based on the altitude minimization of the barycenter. The disk is thus allowed to slide and swivel through a step-by-step process at a random amplitude between zero and a fixed maximum value. After each motion attempt, the position of the particle is rejected if overlapping is detected or if no gain in barycenter altitude is obtained. The present algorithm considers three cycles of 800 attempts for each particle to reach minimum altitude. Between each cycle the maximum amplitudes of translation and rotation are divided by 2 to limit the rejection of movement attempts when reaching the final settled position. Further simulation details are given in [18].

In this study, clay minerals particles are shown as flat rigid disks of diameter *d* = 2 µm and 0.2 µm in thickness, leading to an aspect ratio (i.e., diameter/thickness) of 0.1. This shape is in agreement with the aspect ratio of clay minerals particles experimentally measured by Reinholdt et al. [15] for vermiculite (from 0.08 to 0.10) and by Hassan et al. [37] for illite (from 0.11 to 0.12) and kaolinite (0.07). The width of the square simulation box was set at 8.5*d* to reach a representative elementary volume as demonstrated by Ferrage et al. [18]. Each simulation contains 5500 particles to obtain a disk packing large enough to extract a cubic sub-volume (8.5*d* in width). In order to obtain a wide range of organizations in the final packings, the different simulations were performed considering contrasted initial orientation of the particles (from 0 to 90°) and contrasted maximum amplitude in motion during settling (from *d*/7 to 5*d* and from 0 to 90° for slide and swivel movements, respectively).

**Figure 1:**
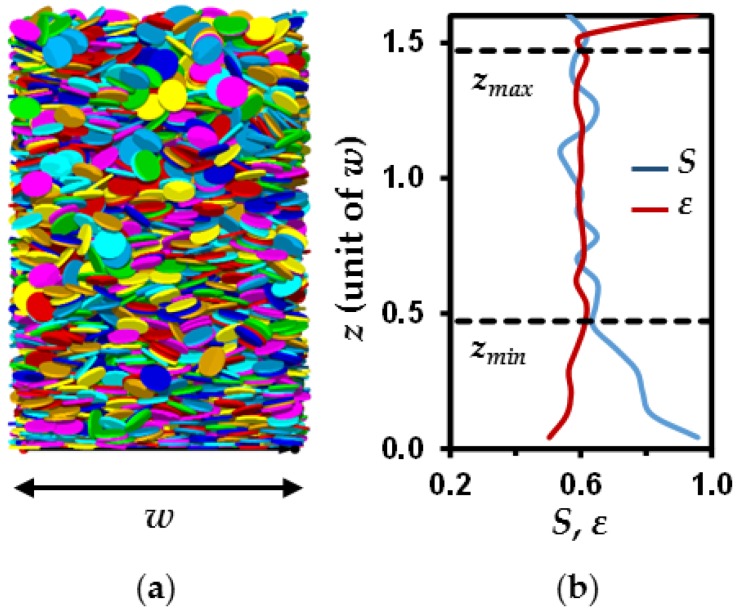
Analysis of porosity and anisotropy in simulated disk packings. (**a**) Three-dimensional packing and (**b**) associated porosity *ε* and order parameter *S* along the *z* direction. The overall porosity and *S* values for the packings are calculated from the nearly homogeneous part of the profiles between *z*_min_ and *z*_max_.

The packings are analyzed in slices along the vertical *z* axis (thickness *dz* and volume *w^2^dz*) allowing for displaying profiles of the porosity and of the orientation of particles. The porosity ε is determined by summing the disk volumes of particles whose barycenter altitude *rz* are found in the range *z* ≤ *rz* < *z* + *dz*. The orientational anisotropy of particles with barycenter comprised of the slice is defined by the order parameter *S* [38,39,40,41,42,43]. A value of 0 describes isotropic systems whereas a value of *S*=1 corresponds to perfectly oriented particles. This order parameter is calculated based on the average of the second-order Legendre polynomial as follows:

(1) $$S=\langle P_{2}\rangle =\langle 3\cos^{2}\theta -1\rangle /2,$$

where *θ* represents the angle between the normal unit vector of the particle and the *z* axis of the simulation box. As illustrated for the packing reported in Figure 1a, the *ε* and *S* parameters are extracted after defining *z_min_* and *z_max_* values corresponding to altitudes delimiting a packing with homogeneous *ε* and *S* profiles (Figure 1b). A denser and more oriented organization is systematically observed below *z_min_* associated with the rigid bottom of the simulation box, leading to particles lying flat on the box surface. Furthermore, packings are more porous above *z_max_* due to incomplete filling of the particle bed with no effect on the orientation of particles. Thus, all values of *ε* and *S* given hereafter were extracted in between carefully chosen *z_min_* and *z_max_* for each medium. An uncertainty of ±0.02 was attributed to both parameters in this study.

### 2.2. Experimental Disk Packings

#### 2.2.1. Experimental Setup and Preparation of Disk Packings

**Figure 2:**
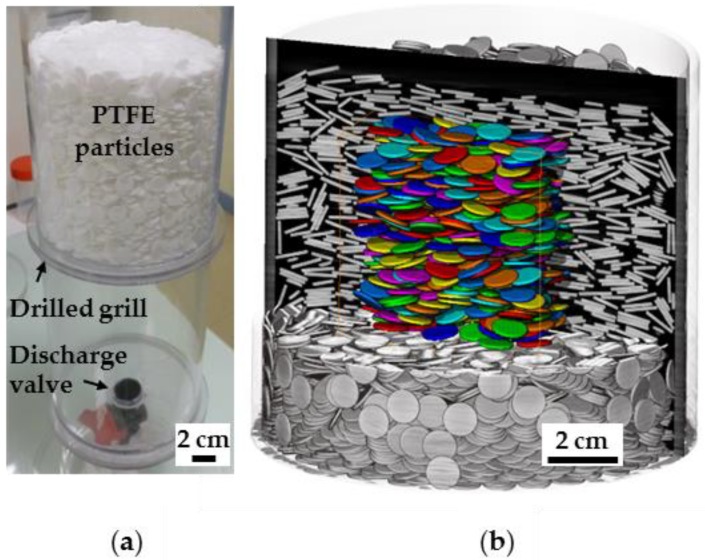
Experimental setup for packing of polytetrafluoroethylene (PTFE) disks. (**a**) Cylindrical column for sedimentation of 10,000 disks in different fluids. Disks are settled in the volume located above a flat drilled grill while evacuation of fluids is achieved through a discharge valve located at the bottom of the column; (**b**) X-ray microtomography analysis and extraction of individual particle orientation in a sub-volume of the experimental packing.

Experiments were performed using Polytetrafluoroethylene (PTFE) disks of aspect ratio = 0.1 to allow a detailed comparison between simulated and experimental data. The disks were designed with a diameter of 1 cm (i.e., 1 mm thickness) as a good compromise between the machining precision on the dimension of the PTFE disks and the resolution on X-ray microtomography images used for *ε* and *S* measurements. About 10,000 disks were allowed to sediment in a poly(methyl methacrylate) (PMMA) cylindrical column of 12 cm diameter (Figure 2a). This sedimentation column is composed of two volumes separated by a drilled grill, both filled by the same fluid. While the top part contains the disks, the bottom part allows for gently evacuating the fluids through a discharge valve without perturbing the particle bed.

To obtain various overall packing organizations, different fluids with various density values were considered in order to adjust the settling rate of particles. A total of 5 experimental disk packings, hereafter referred to as DP1 to DP5, were obtained according to the setting parameters reported in Table 1. DP1 to DP4 differ by the density of the fluids considered from almost 0 (air) to 2.15 (Na-polytungstate) while DP5 has a different particles drop-off strategy. Na-polytungstate is highly soluble in water and thus allows for preparing a controlled density solution depending on its concentration [44,45]. A density of 2.15 for the Na-polytungstate solution represents the maximum value allowing sedimentation of PTFE disks (density of 2.16). Excepted for DP1 where particles were gently deposited on top of the existing bed, for all other DPs, the column was first filled with the selected fluid and the particles were let to settle from a high altitude (typically > 20 cm). From DP1 to DP4 all particles were dropped individually to mimic the one-by-one deposition algorithm with a high value for initial angle. DP5 was prepared as DP4 in a 2.15 density fluid but the particles were dropped all at once, in an attempt to obtain a less anisotropic organization (Table 1). After sedimentation of PTFE disks, liquids were eliminated through the discharge valve and DP3 to DP5 were rinsed 7 times using distilled water to remove Na-polytungstate. Finally, the DPs were dried at room conditions before X-ray microtomographic analysis.

#### 2.2.2. X-Ray Microtomography Analyses

The X-ray microtomographic acquisitions were performed on an EasyTom XL Duo from RX-solutions (Chavanod, France). A sealed microfocus X-ray source L 12161-07 (Hamamatsu Photonics, Hamamatsu, Japan) was used, coupled to a Varian PaxScan 2520DX detector flat panel with amorphous silicon and a CsI conversion screen; 1920 × 1536 pixel matrix; pixel pitch of 127 µm; 16 bits of dynamic (Varian Medical Systems, Palo Alto, CA, USA). The entire samples were scanned with a spatial resolution of 73.36 µm in a helicoid mode (4320 projections in three turns) in order to reduce cone beam. Acquisition parameters were set at 150 kV for tube voltage and 420 µA for tube current. A setting of 12.5 frames per second was used with an averaging of 20 frames per projections. Filtration of the beam was performed using a 1 mm Al filter. The scanning was realized with the large focus mode (i.e., a nominal focal spot size of 50 µm) for a source-to-detector distance of 419 mm and a source-to-object distance of 242 mm. Data reconstruction was achieved using the XAct software v1.1 (RX-solutions, Chavanod, France) with a classical filtered back projection algorithm [46] to correct from beam hardening and ring artefacts. The segmentation of the solid vs. void, porosity, and orientation measurements, as well as visual rendering, were performed on a sub-volume (Figure 2b) using Avizo Software v.9.2 (FEI, Hillsboro, OR, USA) with an adapted image processing methodology mostly based on mathematical morphology tools [47,48]. This latter processing methodology is detailed in the Supplementary Material and accounts for a sequence of data treatments (Figure S1), from the raw image to the segmentation of individual disk particles (Figure 2b).

## 3. Results and Discussion

### 3.1. Evolution of Simulated and Experimental Porosity with Packing Anisotropy

**Figure 3:**
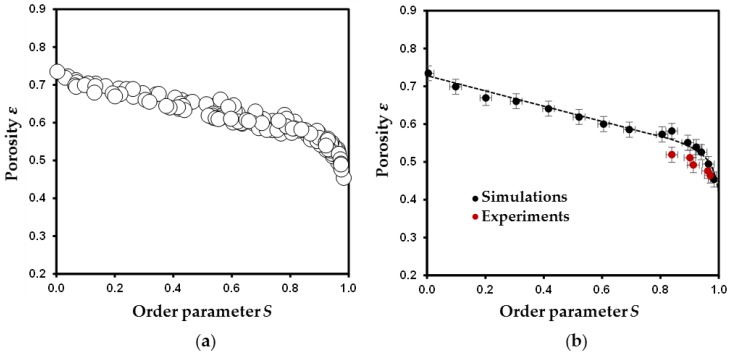
Evolution of porosity *ε* as a function of order parameter *S* for simulated disk packings. (**a**) The 135 disk packings obtained with different degree of freedom in particle motions. (**b**) Selection of 15 disk packings (solid circles) and comparison with experimental porous media (solid red circles). The doted curve highlighting the *ε* vs. *S* master curve is plotted as guideline.

About 135 disk packings were simulated to cover the whole range of anisotropy, i.e., from *S*~0 to *S*~1 (Figure 3a). This was achieved by tuning the degree of freedom in particle motions, based on the input values given to initial angles and maximum amplitudes in swivel or slide motions for particles (see Section 2.1 for the description of the algorithm or [18] for a systematic analysis on the influence of individual parameters on the final packing). As an illustration, low initial angles combined with high maximum amplitudes (i.e., high degree of freedom in particle motions) allow the particle to explore a large number of positions and orientational configurations to minimize its barycenter altitude. This leads to high *S* and low *ε* values whereas, on the contrary, more porous and isotropic packings are obtained when limiting motion amplitudes with high initial angles. As shown in Figure 3a and irrespective of the degrees of freedom given to the particles, all simulated data exhibit the same tendency of decreasing porosity coupled with an increase of the orientational anisotropy (Figure 3a). A change of trend for ε values at *S* > 0.9 is also noticed on this Figure 3a. Although marginal, the dispersion of ε values for a given anisotropy *S* typically results from the role played by individual input parameters on the final packing configurations. As an example, for low degree of freedom in particle motions, the disks can be trapped in local minima leading to the formation of arches structures and to large porosities [49,50,51]. The whole set of data points can be used to confirm the presence of a master curve correlating *ε* and *S* when using this sequential deposition algorithm [18]. This master curve is highlighted in Figure 3b when selecting, from the 135 samples, 15 packings with the lowest *ε* value for a given anisotropy *S*. Input parameters used to obtain these reference packings are reported in Table 2. 

For experimental packings, the extracted *ε* and *S* values are reported in Table 1. The high degree of anisotropy obtained for all packings (0.84 < *S* < 0.97) can be assigned to two principal effects. First, despite the efforts to control the contrast in density between the fluid and the PTFE disks, settling particles were observed to exhibit a low angle value when entering in contact with the existing bed of disks at the bottom of the fluid column. Second, once in contact, the settling PTFE disks were noticed to easily slide on top of other particles, likely associated with the smooth surface of PTFE disks. As pointed out from the simulated packings (Table 2), low degrees of anisotropy (typically for *S* < 0.80) are strongly linked to the initial angle of deposition (typically above 80°) and to the limited extent of slide amplitude (≤*d*). These unreachable conditions in our experiments likely explain the limited range obtained for *S* values (Table 1). Despite this limited extent of anisotropy variation, a negative evolution with *ε* is noticed when increasing *S* (Figure 3b). Furthermore, an overall good agreement is found when comparing experimental and simulated *ε* values for a given *S*. These relatively high *ε* values obtained for both experimental and simulated packings are also consistent with experimental data reported for platy-shaped or tubular clay minerals [43,52,53,54,55]. Note the tendency to have slightly lower experimental *ε* values for porous media prepared in Na-polytungstate fluids (DP3 to DP5) compared to simulations, however. The slightly denser packings obtained experimentally can be first assigned to the fact that PTFE disks were observed to form aggregates through face-to-face contact once immerged in the fluid (DP5) and/or when settling on the bed of particles (DP3 to DP5). As illustrated for DP5, the difference between the experimental *ε* value and that expected to lie on the master curve issued from packing simulations, is of ~0.05 (Figure 3b). This aggregation effect is similar to the sedimentation of disks with higher aspect ratios. As shown by Ferrage et al. [18], an increase of aspect ratio from 0.1 to 0.2 leads to a decrease of ~0.15 in *ε* value for *S*~0.8. Likewise, the work of Ebrahimi et al. [17], using a different algorithm clearly evidenced a significant decrease of porosity coupled with an increase of aggregation. Moreover, X-ray tomographic imagery is a blurry approximation of a true material configuration [56]. Indeed, the structures detectability in tomographic images mainly depends on the inherent resolution limitations of the system and on the partial volume effect (i.e., the value of a voxel covering multiple materials correspond to an average attenuation). Thereby, the smallest pores, such as those located near face-to-face contact of particles in very anisotropic media, are extremely difficult to segment. As a result, a marginal fraction of the smallest pores is omitted and thus slightly lowers the *ε* value. Despite the marginal differences between experimental and simulated *ε* values (Figure 3b), the very good agreement obtained for *S* ≥ 0.84 tends to validate the sequential deposition algorithm used here.

### 3.2. Comparison between Simulated and Experimental Orientation Distribution Functions

The comparison between experimental and simulated data performed above is limited to *ε* and *S* values, which are bulk parameters of the porous media. For instance, different particle organizations can in principle lead to the same macroscopic *S* values. Accordingly, the orientation distribution function (ODF) of particles for experimental and simulated are analyzed and compared below. Because the frame of the simulation box merges with that of the orientation tensor [18], the ODF for disk-shaped particles can be defined here by the function *f*(*θ, ϕ*), where *θ* is angle between the normal of the disk and the *z* axis of the simulation box and *ϕ* the polar azimuthal angle on the (*xy*) plane of the simulation box. For uniaxial systems (cylindrical symmetry) such as those investigated here the ODF is completely described by the function *f*(*θ*) defined as:

(2) f(θ)≥0

and

(3) $$\int_{0}^{\pi }f\left(\theta \right)\sin\theta d\theta =1$$

where sin *θ* accounts for the integration over all azimuthal *ϕ* angles for the solid angle correction [43,57,58].

**Figure 4:**
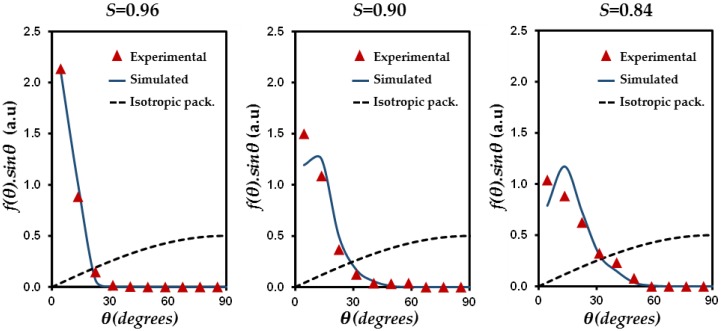
Comparison between simulated and experimental distributions of particle orientation f(θ)sinθ with θ the angle between the normal unit vector of the particle and the z axis of the simulation box. The selected experimental packings correspond to DP1, DP4, and DP5 (with *S* = 0.96, 0.90, and 0.84, respectively, Table 1), whereas simulated packings were chosen in Table 2 for their similar *S* values (with *S* = 0.96, 0.89, and 0.84).

Figure 4 reports *f*(*θ*)*sin* *θ* functions for different *S* values. For comparison with experimental systems (*S* = 0.84; 0.90, and 0.96), simulated porous media with similar *S* values are selected from Table 2 (*S* = 0.84; 0.89, and 0.96). For an isotropic packing (*S* = 0), *f*(*θ*)*sin* *θ* follows a sine behavior. In the case where *S* tends to 1, *f*(*θ*)*sin* *θ* function displays a Dirac-shaped distribution. For similar anisotropy value, it can be shown that the experimental and simulated data show very good agreement, despite the limited number of extracted particles from the experiments (between 671 and 920 particles for experimental media vs. between 3270 and 3955 for simulated packings). This agreement shows that the sequential deposition algorithm used here provides, not only a satisfying reproduction of bulk properties of the experimental porous media, but also, accounts for the details of local particle organization.

### 3.3. Evolution of Geometrical Properties of Simulated Porous Media with Anisotropy

In the following, the 15 simulated porous media (Table 2) are used to get additional information on the evolution of the geometrical properties of the pore network and the solid phase with *S* parameter. This morphological analysis, based bellow on chord length distribution and particle aggregation, is expected to provide a comprehensive understanding of the underlying mechanism at the origin of the observed master curve of *ε* vs. *S* and in particular to the change of porosity trend above *S*~0.9 (Figure 3b). Note that all attempts to apply the same morphological analysis for experimental systems were unsuccessful. This was assigned to the difficulty to detect the smallest pores at the interface between two particles, thus leading to erroneous chord length distribution analyses. 

A chord length analysis provides a morphological description of the solid-pore interface [18,59,60,61,62]. Chords are line segments at the pore-solid interface, for any direction *r*, lying entirely in the pore or solid phase. For the pore phase, the pore chord distribution *f_p_*(*r*) is defined such as *f_p_*(*r*)*dr* is the probability of finding a chord in the pore phase of a length between *r* and *r + dr* and by:

(4) $$\int_{0}^{\infty }f_{p}\left(r\right)dr=1.$$

Based on both the pore chord distribution *f_p_*(*r*) and the solid chord distribution *f_s_*(*r*), the mean chord length for both pore and solid phases along *r* ($l_{p,\bar{r}}$ and $l_{s,\bar{r}}$, respectively) are determined from the first momentum of the distribution function as follows:

(5) $$l_{p,\bar{r}}=\int_{0}^{\infty }r\;f_{p}\left(r\right)dr,$$

(6) $$l_{s,\bar{r}}=\int_{0}^{\infty }r\;f_{s}\left(r\right)dr,$$

where $l_{p,\bar{r}}$ and $l_{s,\bar{r}}$ can be considered to account for the mean sizes of pores and the solid phase along the same direction, respectively. Calculations of pore and solid chord length distributions are performed using 3D voxelized image (512^3^ resolution) for the 15 porous media reported in Table 2.

**Figure 5:**
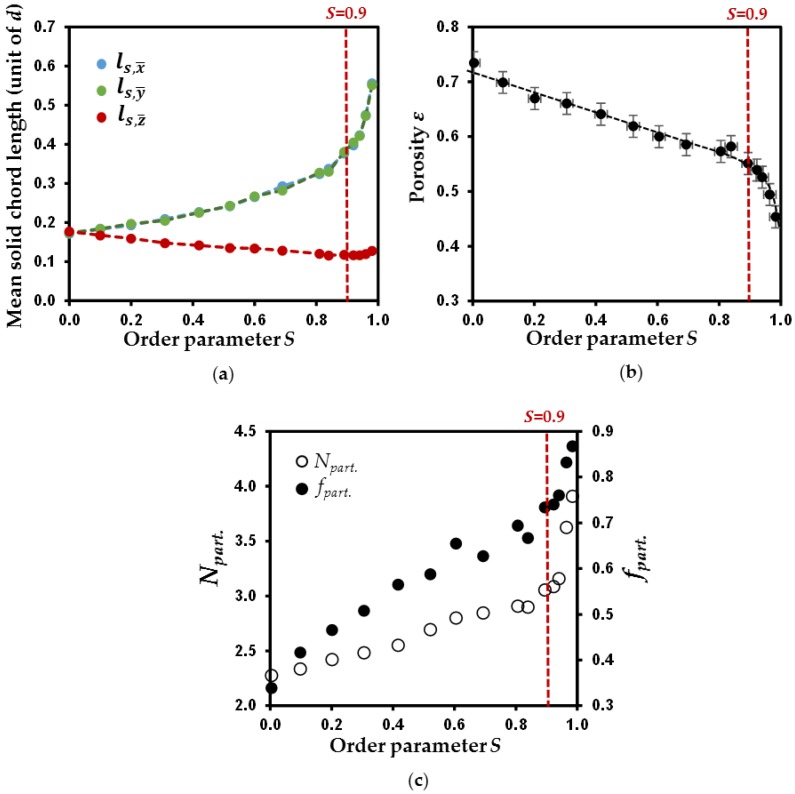
Evolution of geometrical parameters of the simulated porous media as a function of order parameter *S*. Mean chord length for the solid phase in the three main directions x, y, and z (i.e., $l_{s,\bar{x}}$, $l_{s,\bar{y}}$, and $l_{s,\bar{z}}$, respectively) (**a**), porosity *ε* (**b**)*,* and mean number of particles *N_part._* and fraction of particles *f_part._* in aggregates (**c**). Vertical dotted line is a guideline for the change noticed at *S* > 0.9.

The mean chord lengths for the solid phase of the 15 simulated porous media (Table 2) is reported for the three main directions *x*, *y*, and *z* as a function of the order parameter *S* in Figure 5a. Mean chords lengths along the *x* and *y* directions ($l_{s,\bar{x}}$ and $l_{s,\bar{y}}$) are identical, thus confirming the transverse isotropy in simulated packings.

For *S* = 0, the isotropic organization of the packing is corroborated by the equal values for $l_{s,\bar{x}}$, $l_{s,\bar{y}}$ and $l_{s,\bar{z}}$. Figure 5a also reveals that the increase in particle anisotropy leads to a progressive decrease of $l_{s,\bar{z}}$ values and a gradual increase of $l_{s,\bar{x}}$ and $l_{s,\bar{y}}$ values. This evolution is fully consistent with the gradual alignment of particles axes and dimensions with that of the simulation box. Indeed $l_{s,\bar{z}}$ dimension decreases toward a value close to *d*/10 (with *d* the disk diameter), i.e., the thickness of individual disk. Similarly, both $l_{s,\bar{x}}$ and $l_{s,\bar{y}}$ dimensions increase to approach the theoretical mean transverse dimension of the disk at ~0.63*d*. Moreover, note that $l_{s,\bar{z}}$ values slightly increase with *S* when *S* > 0.9 (Figure 5a). This behavior seems to correlate with the abrupt decrease of porosity with *S* increasing for *S* > 0.9 (Figure 5b). Such concomitant increase in $l_{s,\bar{z}}$ dimensions and decrease in *ε* values for *S* > 0.9 could potentially be interpreted by aggregation of particles, defined here by an increased number of face-to-face contact between their flat surfaces leading to an increase of their apparent thickness. The analysis of particle aggregation is thus performed in order to assess its potential influence on the change of porosity for high *S* values. This analysis is performed in a similar fashion as done by Ebrahimi et al. [17,31,32] for the characterization of aggregation of individual nanometer-sized clay layers. The consideration that two particles belong to the same aggregate is based here on three criterions. The first criterion is that the scalar product between the normal vectors associated to the two particles should be larger than 0.95. This ensures the pseudo-parallelism of the two particles surfaces. The second criterion is that the segment formed by the projection of the barycenter position of the first particle onto the surface of the second disk should be shorter than the thickness of the disk. Accordingly, the two particles should first neighbor, while allowing a certain degree of non-parallelism. The third and last criterion is that the distance segment between the two barycenter of the two particles, projected to the surface of one particle, should be shorter than the disk radius. This last criterion allows for a relative lateral displacement between two neighboring particles. While the first criterion is similar to Ebrahimi et al. [17,31,32], these latter authors considered only a second criterion related to the distance between the two particles barycenter. In the case of aggregation of individual clay layers, the consideration of these two criterions leads to the formation of cylindrically stacks of particles with limited lateral misfits, which is fully consistent with the involved cohesive forces [17,31,32]. In the case of aggregation of clay minerals particles constituted by large number of individual layers, contribution from cohesive forces between particles is lowered (or neglected in the present numerical approach), and the aggregates display larger degree of lateral displacements as repeatedly observed here in X-ray microtomographic images. 

**Figure 6:**
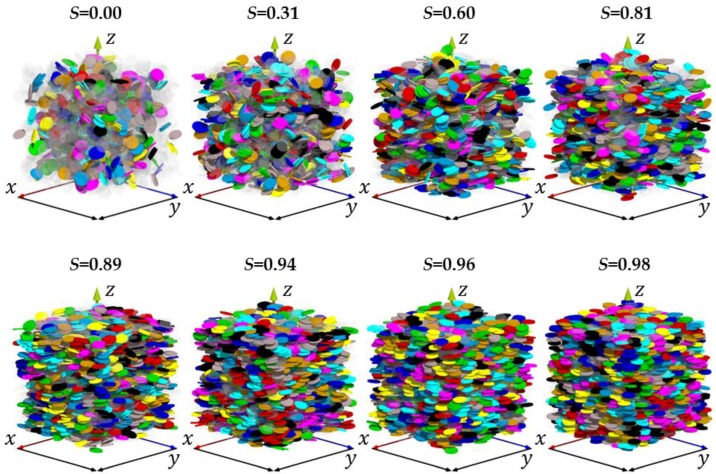
Illustration of particle aggregation for selected simulated packings. A given colour is assigned to all particles from the same aggregate. Translucent particles correspond to disks not involved in aggregates.

The evolution of particle aggregation with *S* parameter, calculated according to the aforementioned methodology for the 15 simulated porous media, is illustrated in Figure 5c. The mean number of particles in the stack, *N_part._* is calculated from the histograms of stack sizes (Table 2). These histograms are found to systematically follow a lognormal-shaped thickness distribution, consistent with different numerical or experimental studies [15,31,32]. In addition, the relative fraction of particles involved in a stack, *f_part._* is also indicated (Figure 5c; Table 2). As seen in Figure 5c and illustrated for selected porous media in Figure 6, both *N_part._* and *f_part._* parameters display a rather monotonic evolution for *S* < 0.9 and then a significant rise for very anisotropic media. This behavior fully supports the hypothesis that aggregation is of the origin of the abrupt decrease of porosity with an increase of *S* when *S* > 0.9 (Figure 5b).

If particle orientation and aggregation can be used to interpret the evolution of *ε* with *S*, analysis of chord distribution can also be used to derive quantitative indicators of the anisotropy of the solid and pore phases. This can be done by calculating ratios between mean chord length along the *z* and *xy* directions as:

(7) $$R_{p}=l_{p,\bar{z}}/l_{p,\bar{xy}},$$

(8) $$R_{s}=l_{s,\bar{z}}/l_{s,\bar{xy}}.$$

**Figure 7:**
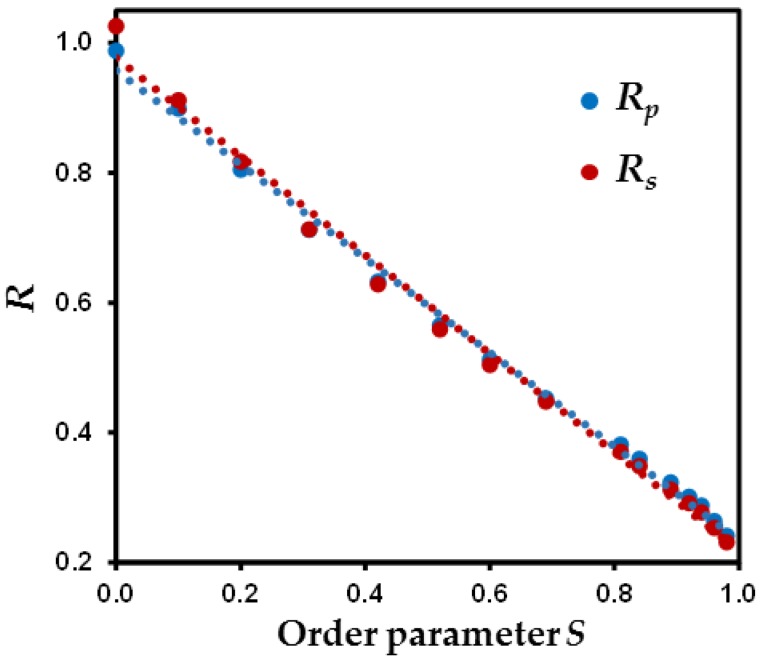
Evolution of ratios between mean chord length between the *z* and *xy* directions for both the solid and pore phases (*R_s_* and *R_p_*, respectively) as a function the order parameter *S*.

Evolution of *R_p_* and *R_s_* parameters with *S* parameter reported in Figure 7 evidences that anisotropy in the pore or solid phase are fairly similar. These anisotropy indicators range from nearly 1 when *S* = 0 to ~0.2 for the most anisotropic medium and show a surprising linear correlation with *S* (Figure 7). This finding suggests that extraction of *S* parameter, easily derived from experimental techniques such as diffraction methods [36,41,43,63,64,65], can potentially be used to extract information regarding anisotropy in the pore network, this latter being difficult to obtain experimentally.

## 4. Conclusions

Simulation of 3D disk packings is an efficient approach to deepen our understanding of the role played by anisotropy in orientation of flat particles on the geometrical properties of the whole porous medium. This is particularly relevant in the case of compacted anisometric particles which can display a wide range of *S* values [18,41,43].

In this study, non-interacting disk packing simulations confirmed the close relationship between porosity and anisotropy through the *ε* vs. *S* master curve. Although limited to very anisotropic systems (for *S* > 0.8), experiments also validated the obtained correlation. Additional analyses of evolution of geometrical parameters with anisotropy of the porous media demonstrated that the significant decrease of porosity for *S* > 0.9 was associated to both particle orientation and particle aggregation. Interestingly this aggregation is noticed here even though no interaction forces are considered in between particles during the simulation of settling process.

Morphological analyses of the porous media through chord length measurements show that *R_p_* and *R_s_* parameters fall onto a linear correlation with the order parameter *S*. This relation is particularly relevant for correlating the orientational properties of particles easily accessible experimentally with anisotropy in the pore network. In this regard, the logical perspective of this work is to analyze the influence of *S* parameter on the anisotropy of diffusional properties of water in the porous medium. 

## Figures and Tables

**Table 1:** Settling conditions (density of fluid and particle drop-off procedure chosen) for disk packing experiments and associated extraction of porosity *ε* and order parameter *S* (uncertainty of ±0.02).

Sample	DP1	DP2	DP3	DP4	DP5
Fluid	Air	Water	Na-polytungstate	Na-polytungstate	Na polytungstate
Density	10^−3^	1.00	2.10	2.15	2.15
Drop-off	Individual	Individual	Individual	Individual	All at once
*ε*	0.48	0.47	0.49	0.54	0.51
*S*	0.96	0.97	0.91	0.90	0.84

**Table 2:** Algorithm parameters used to generate the selected 15 simulated disk packings. For each medium, all particles are given an initial angle (in degree) and maximum amplitudes motions to swivel (in degree) and to slide (relative to particle diameter *d*) during the settling process. The parameters *S* and *ε* stand for the order parameter and the porosity, respectively (uncertainty of ±0.02). *N_part._* and *f_part._* represent the mean number of particles and the fraction of particles in aggregates, respectively.

Initial Angle	Max. Swivel Amplitude (°)	Max. Slide Amplitude (µm)	*S*	*ε*	*N_part._*	*f_part._*
82	11	*d/*6.4	0.00	0.73	2.28	0.34
87	22	*d/*7	0.10	0.70	2.33	0.42
87	25	*d/*2	0.20	0.67	2.42	0.47
85	40	*d/*7	0.31	0.66	2.48	0.51
89	50	*d/*7	0.42	0.64	2.55	0.56
82	60	*d/*2	0.52	0.62	2.69	0.59
85	85	*d/*3	0.60	0.60	2.80	0.65
85	80	*d*	0.69	0.59	2.85	0.63
60	60	*d*	0.81	0.57	2.91	0.69
47	28	*d/*3	0.84	0.58	2.90	0.67
40	40	*d*	0.89	0.55	3.06	0.73
25	45	*d/*2	0.92	0.54	3.09	0.74
18	55	*d*	0.94	0.53	3.16	0.76
10	70	*4d*	0.96	0.49	3.63	0.83
0	80	5*d*	0.98	0.45	3.91	0.87

## Supplementary Materials

This Supplementary Material details the processing methodology used in this article to segment individual disk particles from raw X-ray microtomographic images. It is available online at http://www.mdpi.com/1996-1944/11/10/1972/s1, Figure S1: Processing methodology for porosity measurement and extraction of individual particle orientation (see text for details): (**a**) Non-local mean image (central vertical section in the sub-volume), (**b**) gradient image, (**c**) segmentation result of porosity vs. disks, (**d**) erosion of c, (**e**) distance map of d, (**f**) segmentation of e, (**g**) subtraction off from d, (**h**) erosion with a diskoid structuring element, (**i**) labelled markers, (**j**) watershed transformation of the gradient image b using the markers in i, (**k**) final result after manual correction of markers and quantitative filtration, (**l**) 3D rendering of the final result.
