# Supplementary material for: Mesoscale Anisotropy in Porous Media Made of Clay Minerals. A Numerical Study Constrained by Experimental Data
Source: Materials (Basel). 2018 Oct 13;11(10):1972. doi: 10.3390/ma11101972 (PMC6212813; doi:10.3390/ma11101972)
Supplement: Supplementary file 1 [file materials-11-01972-s001.pdf]

materials

MDPI

Supplementary Material

Mesoscale Anisotropy in Porous Media made of Clay Minerals. A Numerical Study Constrained by Experimental Data

Thomas Dabat *, Arnaud Mazurier, Fabien Hubert, Emmanuel Tertre, Brian Grégoire, Baptiste Dazas and Eric Ferrage *.

Université de Poitiers, CNRS, UMR 7285 IC2MP, Equipe Hydrasa, 5 rue Albert Turpain, Bâtiment B8, TSA 51106, 86073 Poitiers Cedex 9, France.

* Correspondence: thomas.dabat@univ-poitiers.fr; eric.ferrage@univ-poitiers.fr; Tel.: +33-549-366-395.

This Supplementary Material details the processing methodology used in the corresponding article to segment individual disk particles from raw X-ray microtomographic images.

1. Porosity measurement

According to the grey levels variations across particles resulting from some artefacts (*e.g.* partial volume effect, and cone beam artefact) and the possible residual presence of water or Na polytungstate intercalate between the PTFE discs, a simple thresholding by boundary of the X ray microtomographic data would have provide erroneous discrimination of voxels. Therefore, the segmentation of reconstructed slices for porosity measurements have followed an image processing routine detailed below and apply under the software Avizo v.9.2 (FEI):

(1) Extraction of a cubic sub-volume of interest of 818x818 voxels (*i.e.* side length of ~60 mm) in the centre of each column in order to avoid edge effects along the PMMA container wall.
(2) Attenuation of high-frequency noise while preserving the edge of structures with a windowed non-local means filter (NLM; [1] (Figure S1a).
(3) Coarse thresholding by boundary to defined two sets of markers: one for the PTFE discs and one regrouping air, water and eventually Na-polytungstate residuals. The thresholding leave unlabelled regions which correspond to portions of different phases having same grey levels.
(4) 3D shrinking of the markers by three successive erosions of one voxel.
(5) Watershed segmentation [2] based on the markers and the NLM gradient image computed in 3D (Figure S1b). The unlabelled volumes are filled by flooding from the markers until the phase boundaries enhanced in the gradient image (i.e., the landscape gradient image controls the expansion of markers).
(6) Segmentation of the step-5 result through a black top-hat (i.e., the NLM image subtracted from its morphological closing) in order to remove voxels corresponding to small interstices between packed/agglutinated discs. Those elements represent small volumes, filled by air or solutions, subject to the partial volume effect and then have grey levels of intermediate value between PTFE and Air/Na-Polytungstate. The result is presented in Figure S1c.

The volumetric ratio between porosity and the discs is then computed to derive ◎ values.

2. Assessment of individual particles orientation

In order to calculate the order parameter *S*, particles need to be individualized together with their own orientation frame. The difficulty of this procedure results from the fact that the discs, which have same grey levels, can exhibit numerous contacts, up to the superposition of several particles forming aggregates. The discrimination of individual particles is then obtained according to the following steps:

(7) 3D shrinking of discs segmented at the step-6 by four successive erosions of one voxel. This notably allows to disconnect the discs with weak contact by the edges or weak superposition (Figure S1d).
(8) Computation of a Euclidean distance map of the thinned discs: each voxel will be assigned a grey level value depending on the distance to the nearest object boundary (Figure S1e).
(9) Segmentation by boundary of the distance map: the voxel with high grey levels, which correspond to superposition part of the discs aggregate, are selected (Figure S1f) and subtracted from the results of step-7 (Figure S1g).
(10) Erosion with a structuring element matching with a disc in order to remove the connections remaining between the discs of the aggregates (Figure S1h).
(11) Labelling of each elements resulting from step-10 (Figure S1i).
(12) Flooding from the labelled markers defined in step-11 with the gradient map (Figure S1b) as landscape image (marked based watershed) (Figure S1j).
(13) Quantitative filtration: a group of measures is computed on each component resulting from step-11 in order to remove the elements which present a morphology far distinct from a PTFE disc, i.e., mostly those which are still agglutinated or on the edge of the volume. The computed parameters and their definition according to the software Avizo, as well as the followed selection, are as follow:
   - *Length3d* : maximum of the Feret Diameters; elements superior to 11 mm have been removed;
   - *Volume3d* : volume of the element; elements superior to 95mm^3 and elements inferior to 75mm^3 have been removed;
   - *Anisotropy*: 1 minus the ratio of the smallest to the largest eigenvalue of the covariance matrix. Measures a region’s deviation from a spherical shape. Flat objects have values close to 1; elements with Anisotropy inferior to 0.98 have been removed;
   - *BorderVoxelCount*: some discs might be intersected by the border of the image volume. The computed value for each element corresponds to the voxels number that are touching the border. Objects with non-zero value are removed.
(14) Manual correction of labelled markers corresponding to the rejected elements.
(15) The step-12 is throwing again with corrected labelled markers.
(16) The step-13 is throwing again and elements which still do not meet the previous conditions (Figure S1k), mostly the ones touching the border, are rejected. At this step, the results is satisfying even if some segmented discs present a rough surface in 3D rendering (Figure S1l).

For each segmented disc, the coordinate of the eigenvector 3, normal to the surface of the disc, is computed and used to calculate the parameter *S*.

Reference

1. Buades, A.; Coll, B.; Morel, J.-M. A non-local algorithm for image denoising. In; IEEE, 2005; Vol. 2, pp. 60–65.
2. Roerdink, J. B.; Meijster, A. The watershed transform: Definitions, algorithms and parallelization strategies. *Fundam. Informaticae* **2000**, *41*, 187–228, doi:10.3233/FI-2000-411207.

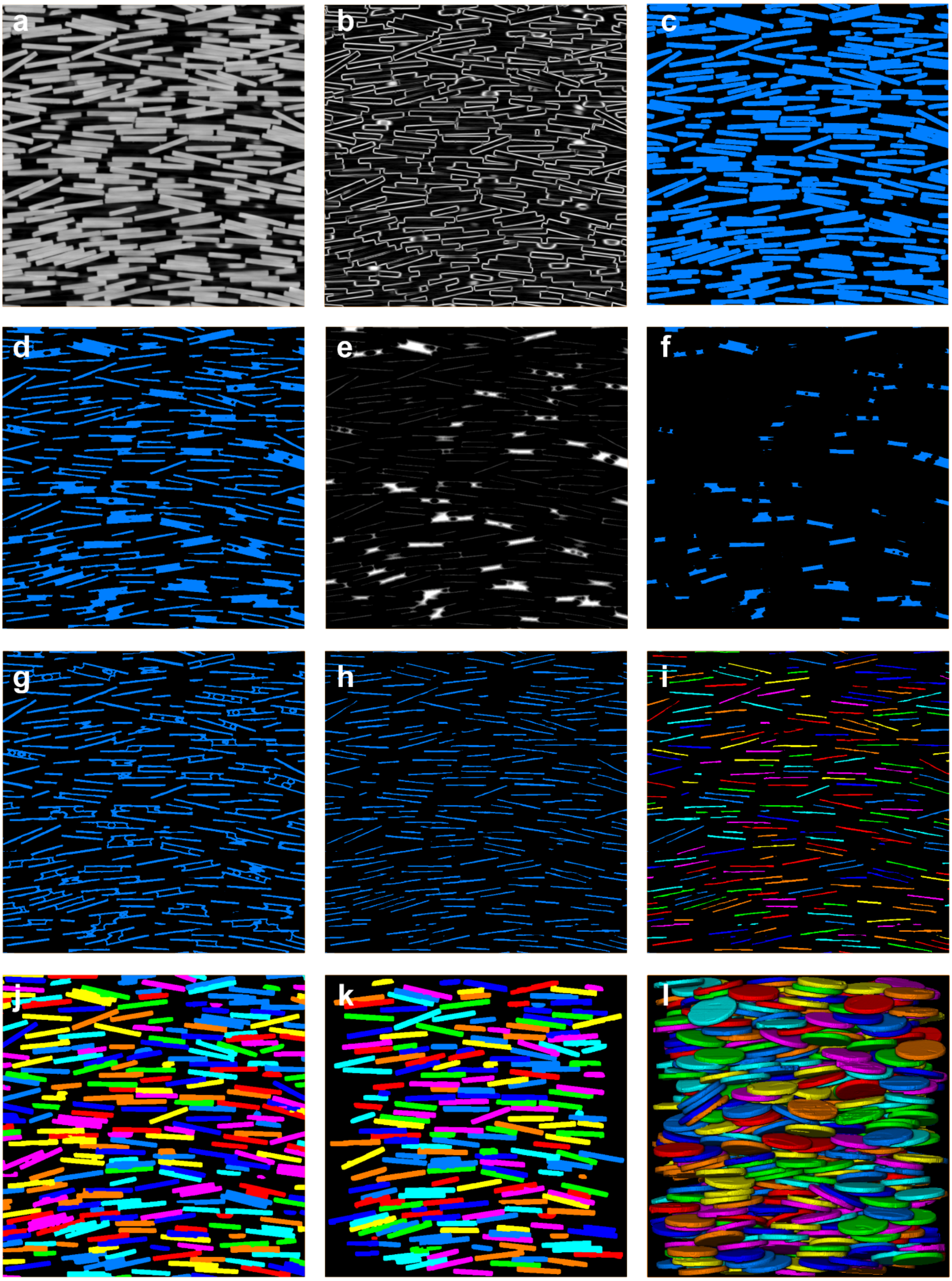

Figure S1. Processing methodology for porosity measurement and extraction of individual particle orientation (see text for details): (**a**) Non-local mean image (central vertical section in the sub-volume), (**b**) gradient image, (**c**) segmentation result of porosity vs. discs, (**d**) erosion of c, (**e**) distance map of d, (**f**) segmentation of e, (**g**) subtraction off from d, (**h**) erosion with a discoid structuring element, (**i**) labelled markers, (**j**) watershed transformation of the gradient image b using the markers in i, (**k**) final result after manual correction of markers and quantitative filtration, (**l**) 3D rendering of the final result.
